# A novel missense *TGFBI* variant p.(Ser591Phe) in a Finnish family with variant lattice corneal dystrophy

**DOI:** 10.1177/1120672121997305

**Published:** 2021-03-01

**Authors:** Aino Maaria Jaakkola, Petri J Järventausta, Reetta-Stiina Järvinen, Pauliina Repo, Tero T Kivelä, Joni A Turunen

**Affiliations:** 1 Department of Ophthalmology, University of Helsinki and Helsinki University Hospital, Helsinki, Finland; 2 Folkhälsan Research Center, Biomedicum Helsinki, Helsinki, Finland

**Keywords:** Corneal dystrophy, lattice corneal dystrophy, amyloid, TGFBI, pathogenic variant

## Abstract

**Introduction:**

We describe the phenotype of a variant lattice corneal dystrophy (LCD) potentially caused by a novel variant c.1772C>T p.(Ser591Phe) in exon 13 of the transforming growth factor beta-induced *(TGFBI)* gene.

**Case report:**

The proband, a 71-year-old woman referred because of bilateral LCD, first seen at the age of 65 years, with recent progressive symptoms, underwent a clinical ophthalmological examination, anterior segment optical coherence tomography and confocal microscopy. Additionally, three siblings and three children were examined. The identified *TGFBI* variant was screened in six family members using Sanger sequencing. A corneal dystrophy gene screen was performed for the proband. Translucent subepithelial irregularities and central to midperipheral stubby branching corneal stromal lattice lines, asymmetric between the right and the left eye, were visible and resulted in mild deterioration of vision in one eye. Genetic testing revealed a novel variant c.1772C>T in *TGFBI*, leading to the amino acid change p.(Ser591Phe). One daughter carried the same variant but had only thick stromal nerve fibres at the age of 49 years. The other family members neither had corneal abnormalities nor carried the variant. No keratoplasty is yet planned for the proband.

**Conclusions:**

We classify the novel variant in *TGFBI* as possibly pathogenic, potentially causing the late-onset, asymmetric variant LCD. Our findings add to the growing number of *TGFBI* variants associated with a spectrum of phenotypes of variant LCD.

## Introduction

Classic lattice corneal dystrophy (LCD) and its variants are one of the most frequently encountered corneal dystrophies, consisting of a group of inherited disorders characterized by a lattice-like accumulation of amyloid in the corneal stroma, associated with recurrent erosions, stromal ground-glass haze and eventually subepithelial scarring.^1^ It is often but not always bilateral, and may be asymmetric. LCD is caused by pathogenic variants in the transforming growth factor beta -induced (*TGFBI*) gene.

In its classic form (LCD1; OMIM #122200), which is caused by definition by the *TGFBI* variant p.Arg124Cys, amyloid deposits appear at an early age and lead to gradual bilateral opacification of the cornea.^1,2^ Visual impairment typically requires corneal transplantation after the fourth decade. To date, more than 40 different variants in *TGFBI* have been reported to cause a variant LCD. Initially, an effort was made to name them (e.g. LCD type IIIA, I/IIIA, IV), but depending on the mutated codon and the age of the patient their phenotypes not only differ but also overlap each other.^3^ In the IC3D classification of corneal dystrophies, they are all simply labeled ‘variant’.^1^ A variant LCD can have a delayed onset, it may progress in an asymmetric manner, and the distribution and size of lattice lines may vary.^1^ The clinical findings depend on the pathogenic variant and the age of the patient, they vary between families carrying the same variant, and the penetrance may also differ.

We report the phenotype of a variant LCD potentially caused by a missense variant c.1772C>T in the *TGFBI* gene, leading to the amino acid change p.(Ser591Phe) in a Finnish family. This variant has not been reported earlier, suggesting it may be a novel pathogenic variant that may lead to a late-onset, asymmetric variant LCD.

## Case description

The 71-year-old proband had been clinically suspected of having LCD at the age of 65 years, at which time she had full vision and no symptoms, whereas no corneal abnormalities had been seen at the age of 53 years. She had no family history of corneal disease, ocular trauma, or familial amyloidosis, Finnish type.^4^ During the past year, her visual acuity declined, ocular discomfort appeared and a spontaneous corneal erosion developed in her right eye. The proband was enrolled after being referred to the Cornea Service, Department of Ophthalmology, Helsinki University Hospital, Finland. She underwent a thorough clinical examination, including best corrected visual acuity (BCVA), tonometry, corneal sensation testing using a cotton-tipped applicator, biomicroscopic evaluation and anterior segment optical coherence tomography (AS-OCT), and corneal confocal microscopy. Her BCVA was 0.9 and 1.0 in her right and left eye, respectively. The keratometric values were 41.9/41.6 D (steep/flat) in her right, and 42.5/41.8 D in her left eye. Slit lamp examination revealed an inferonasal corneal erosion and corneal stromal lattice lines beneath and around the erosion in her right eye, and a few lattice lines in the inferior midperipheral left cornea. Bilateral corneal arcus was noted. The corneal endothelium, anterior chamber, iris and intraocular pressure were normal. She had a mild age-related cataract. The corneal erosion healed with minor scarring. Corneal sensation was normal in both eyes.

**Figure 1.**
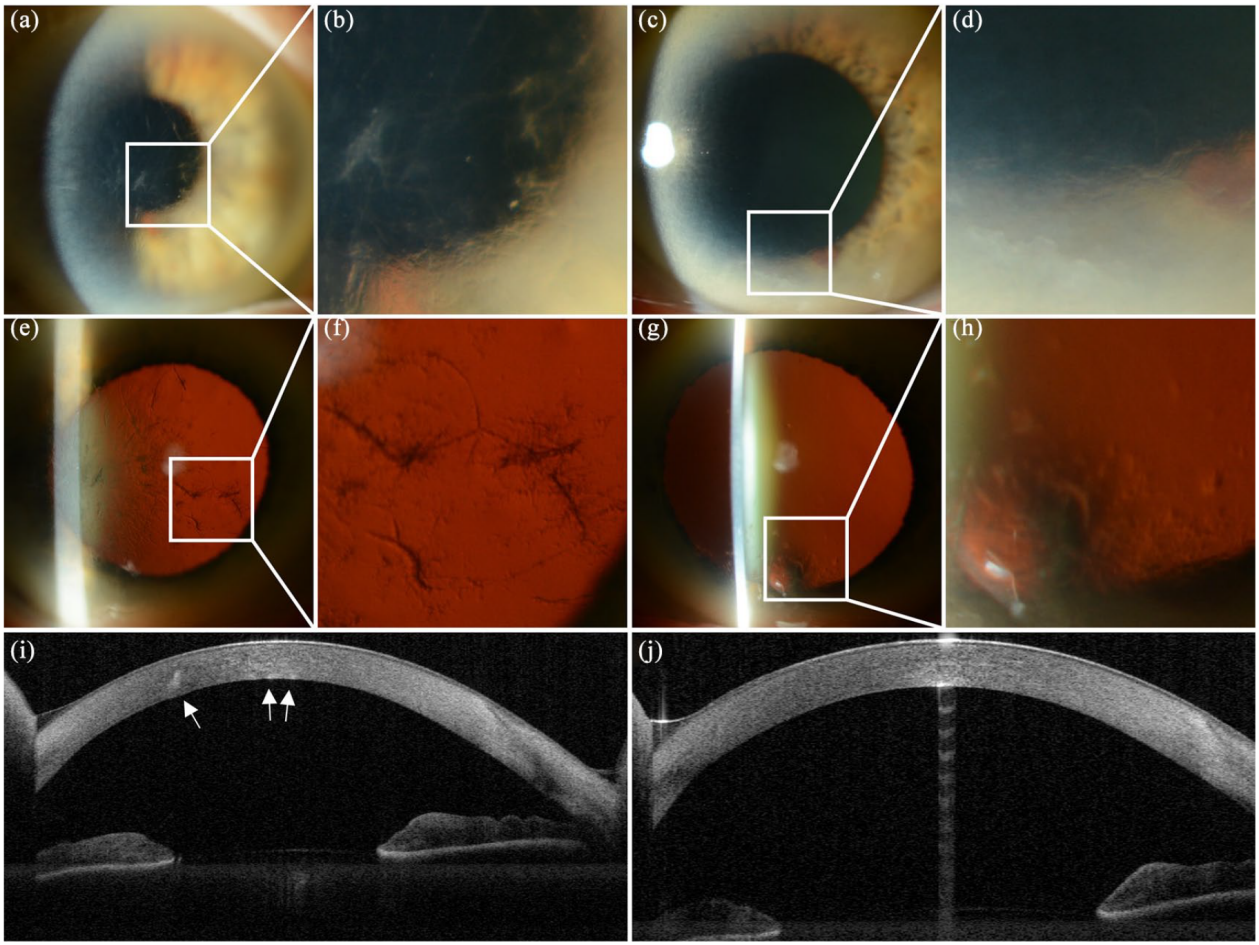
Anterior segment photography and optical coherence topography (AS-OCT) of the proband (II.2). Translucent subepithelial irregularity and stubby branching stromal lattice lines in the right eye (a, b) and similar localized opacities and faint lattice lines in the lower part of the left cornea (c, d). Corneal retroillumination of the right eye (e, f) and left eye (g, h) highlights the deposits. AS-OCT of the right eye shows hyperreflective deposits in the corneal stroma (i) whereas the left cornea is unremarkable (j).

**Figure 2.**
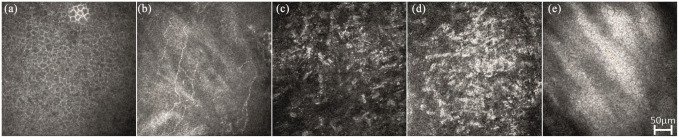
Corneal confocal images of the right eye of the proband (II.2). The epithelium (a) and the subepithelial nerves (b) are interpreted as normal. Stromal opacities involve mainly the middle (c) and posterior stroma (d). Hyperreflective haze is interpreted as possible stromal scarring. The endothelium is normal (e).

**Figure 3.**
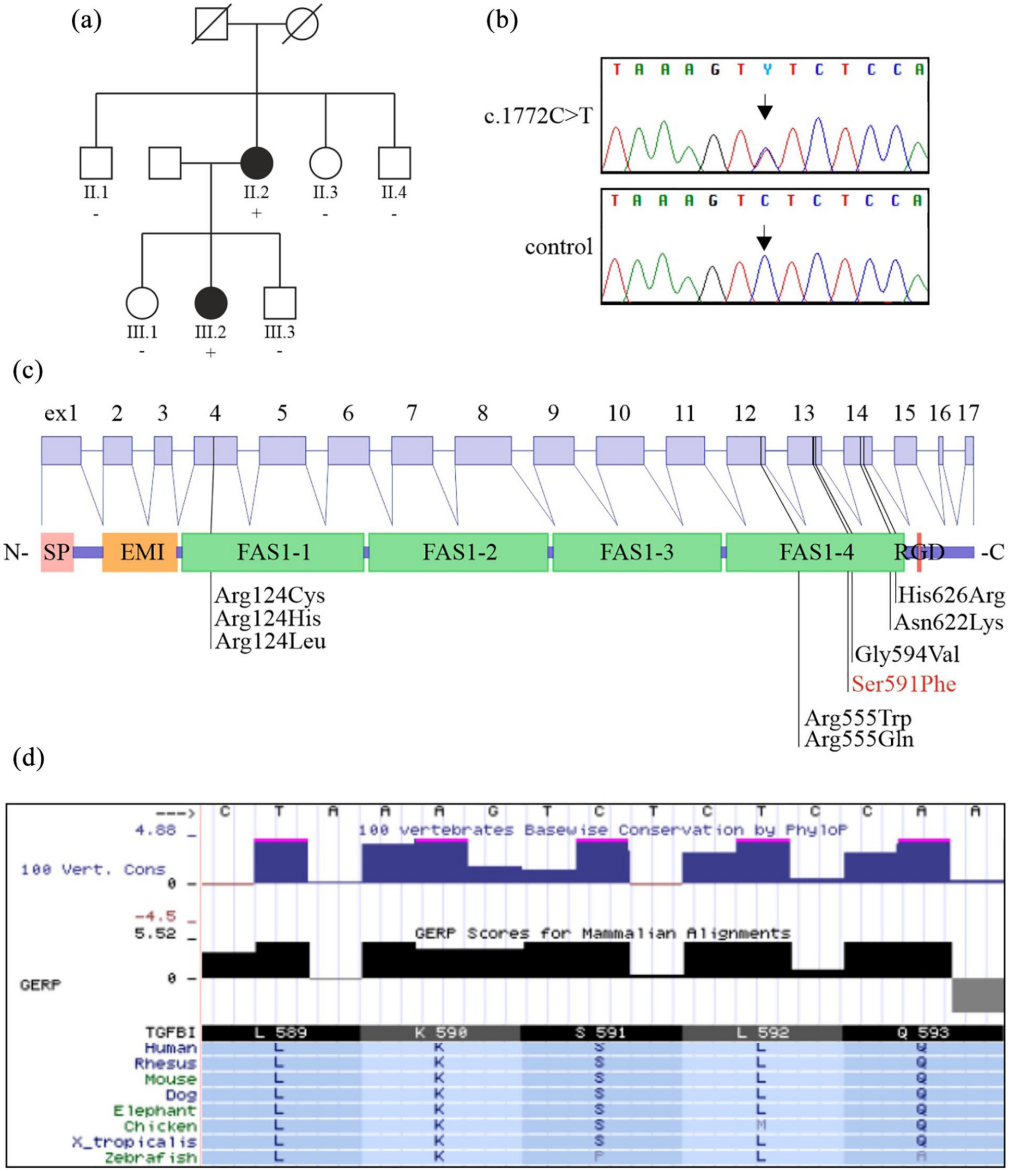
The pedigree of the family (a). Individuals with an ID number were examined. Plus sign indicates the presence and the minus sign the absence of the *TGFBI* variant c.1772C>T. Sequence chromatogram of the c.1772C>T variant for the proband and for a non-carrier family member (b). The structure of the *TGFBI* gene and protein with the localization of the most prevalent TGFBI pathogenic variants, including Ser591Phe (c). Other asymmetric or late-onset lattice dystrophy-associated variants Gly594Val, Asn622Lys, and His626Arg are also shown. The serine residue at position 591 is conserved across species (d; data from the University of California Santa Cruz (UCSC) genome browser).

A year later, her BCVA was unchanged. Slit lamp examination of the right eye showed translucent subepithelial irregularity, relatively short and thick, branching centrally located lattice lines (Figure 1(a) and (b)), which extended midperipherally, especially inferotemporally. Similar, inferiorly located subepithelial irregularity and fainter lattice lines were noted in her otherwise normal left eye (Figure 1(c) and (d)). Retroillumination highlighted the translucency of the subepithelial and stromal deposits (Figure 1(e)–(h)). AS-OCT of the proband showed hyperreflective deposits in the corneal stroma, mainly in the right eye (Figure 1(i) and (j)). Central corneal thickness was 559 µm in the right eye and 543 µm in the left eye. Specular microscopic imaging showed a normal endothelial cell morphology and density. Corneal confocal microscopy of the right eye (Figure 2) demonstrated stromal opacities involving mainly the middle and the posterior stroma. Opacities varied from small hyperreflective dots to larger irregular areas. Hyperreflective haze, possibly representing stromal scarring, was also noted. The corneal epithelium and endothelium appeared normal. We failed to capture the larger lattice lines. Two of her brothers, one sister, two daughters and one son were examined (Figure 3(a); Table 1). A 49-year-old daughter (III.2) with BCVA 1.0 had bilateral thickening of stromal nerves. None of the other family members had any relevant corneal findings.

**Table 1.** Clinical findings in a Finnish family with variant lattice corneal dystrophy.

Individual	Sex	Age	Eye	BCVA	Refraction	IOP	Corneal findings
II.1	Male	75	R	1.0	0+0.75a × 65	11	Corneal arcus, bilateral chronic blepharitis
			L	1.0	−0.25+0.75a × 115	12	
II.2	Female	72	R	0.9	+3.00−1.00a × 100	19	Subepithelial irregularity, stubby branching lattice lines, corneal arcus, normal corneal sensation, spontaneous erosion
			L	1.0	+2.25−0.50a × 100	15	Focal subepithelial irregularity and faint lattice lines in inferior midperiphery, clear central cornea, corneal arcus, normal corneal sensation
II.3	Female	67	R	1.0	+1.25−0.50a × 155	13	Bilateral corneal arcus, delicate crocodile shagreen
			L	1.0	+1.75−1.00a × 80	13	
II.4	Female	63	R	1.0	+0.75+0.50a × 10	14	Bilateral corneal arcus, bilateral chronic blepharitis
			L	1.0	0+0.50a × 0	15	
III.1	Female	51	R	1.0	+1.00−0.50a × 60	18	Bilateral dry eye, a few prominent nerve fibres superiorly, corneal iron line
			L	1.0	+1.00−0.50a × 120	18	
III.2	Female	49	R	1.0	emmetropia	19	Bilateral prominent nerve fibres, corneal iron line
			L	1.0	emmetropia	21	
III.3	Male	41	R	1.0	emmetropia	15	Slight haze in inferior anterior stroma of the right eye, bilateral corneal iron line, prominent nerve fibres and corneal arcus
			L	1.0	emmetropia	17	

BCVA: best corrected visual acuity; IOP: intraocular pressure.

Blood samples were collected from the proband and family members to obtain DNA. Genetic analysis in the proband included polymerase chain reaction (PCR) amplification and Sanger sequencing of the most frequently mutated exons 4, 11, 12, 13 and 14 of *TGFBI* as well as the gelsolin (*GSN*) pathogenic variant c.640G>A, which in Finland causes familial amyloidosis, Finnish type (FAF) with corneal lattice amyloidosis,^5^ using standard HUSLAB protocols. Exome sequencing (ES) was performed at the Institute for Molecular Medicine Finland (FIMM, Helsinki, Finland) as previously described.^6^ In silico predictions were performed with PolyPhen,^7^ SIFT,^8^ MutationTaster^9^ and CADD.^10^ Analysis of conserved sequences was obtained from the University of California Santa Cruz (UCSC) genome browser (http://genome.ucsc.edu/) using the human genome build h37/hg19.^11^ Variant allele frequency was examined using the gnomAD r2.0.2 database (http://gnomad.broadinstitute.org), accessed June 2019.^12^ The location of the fourth fasciclin 1 (FAS1-4) domain of TGFBI (Q15582) was determined using the Uniprot database.^13^ Genetic testing of the index patient revealed wild type *GSN* and a variant c.1772C>T (Figure 3(b)) in exon 13 of the *TGFBI* gene that leads to the amino acid change p.(Ser591Phe) (Figure 3(c)). Multiple prediction programs predict it to be pathogenic (Polyphen2: probably damaging, SIFT: damaging, MutationTaster: disease causing, scaled-CADD score: 34). The variant has not been reported before, and it is not present in the gnomAD database that includes 12,897 Finns. The corresponding serine is highly conserved across species, further supporting a pathogenic effect for this variant (Figure 3(d)). Because paraproteinemic keratopathy associated with monoclonal gammopathy can mimic LCD,^14^ we performed serum protein immunoelectrophoresis with normal findings. Finally, to exclude other possible corneal dystrophies, we performed ES and candidate gene analysis for the proband. After filtering, we detected two rare heterozygous variants: the already known c.1772C>T in *TGFBI* and c.6457G>A, p.(Val2153Ile) in *ZNF469*. Recessively inherited pathogenic variants in *ZNF469* cause brittle cornea syndrome 1 (BCS1; OMIM #229200), characterized by extreme thinning of the cornea and sclera. The *ZNF469* c.6457G>A variant was predicted to be benign by all prediction programs used. Therefore, we conclude that this variant is very unlikely the cause of this variant LCD phenotype. We sequenced the variant c.1772C>T in *TGFBI* from the six family members. The 49-year-old daughter (III.2) with bilateral thickening of stromal nerves had the same variant as the proband. None of the other family members carried the variant (Figure 3(a)).

## Conclusions

We are unaware of a previous report of corneal dystrophy associated with the p.(Ser591Phe) variant in *TGFBI*. Compared with classic LCD, deterioration of vision in the proband began much later.^3^ In addition to the proband, who had mild visual disturbance at the age of 71 years and visible lattice lines 7 years earlier, we found that the only relative who carried the same variant had mildly thickened corneal nerves at the age of 49 years. This could be an incidental finding or a very early stage of lattice dystrophy.

Considering all evidence, we propose that the variant c.1772C>T may cause the late-onset, asymmetric variant LCD in our proband. We predict that the daughter carrying the same variant will develop lattice lines and, later, symptoms and we have recommended follow-up in her case. The proband still has very good vision 8 years after the lattice lines were first seen, suggesting that this variant not only appeared late but also progressed slowly thereafter, and no corneal surgery has been scheduled for her yet. Although she has suffered from a superficial corneal erosion at least once, her corneal sensation was normal, and recurrent erosions have not been her leading symptom, in contrast to classic LCD.

The location of the pathogenic amino acid changes in TGFBI affects the phenotype of corneal dystrophies. The p.(Ser591Phe) variant is located in the FAS1-4 domain. Interestingly, pathogenic variants in codon 555 in this domain, Arg555Trp and Arg555Gln, cause granular corneal dystrophy type 1 (GCD1) and Thiel-Behnke corneal dystrophy (TBCD), respectively, which are characterized by non-amyloid deposits. The classic LCD pathogenic variant Arg124Cys is located in the FAS1-1 domain. Variants such as Gly594Val near Ser591Phe, Asn622Lys and widely diagnosed His626Arg are associated with an asymmetric or a late-onset variant LCD.^3^

Because only one, relatively young sibling carried the variant, and we were unable to reach more distant, older family members, we cannot completely exclude the possibility that the variant LCD phenotype is caused by another mutation than the p.(Ser591Phe) variant, although we consider such a possibility unlikely. To definitively prove that this variant is causing the dystrophy, we would need to find new patients who carry the same variant and share the phenotype, or perform functional in vitro studies. Nevertheless, the bilateral dystrophy of the proband, its late onset as compared with the age of the unaffected carrier daughter, bioinformatics, conservation of the Ser591 residue, and its location in the FAS1-4 domain, all point to the p.(Ser591Phe) variant as the cause of the late-onset, asymmetric variant LCD in this Finnish family. The fact that many different variants in *TGFBI* can cause overlapping phenotypes highlights the importance of genetic analysis in reaching the correct diagnosis.
